# From hysteria to conversion: A case of stuttering

**DOI:** 10.1192/j.eurpsy.2021.495

**Published:** 2021-08-13

**Authors:** R. Silva, J. Camilo, I. Vaz, A.M. Ribeiro

**Affiliations:** 1 Centro De Gestão De Psiquiatria E Saúde Mental, Centro Hospitalar de Trás-os-Montes e Alto Douro, Vila Real, Portugal; 2 Psiquiatria, Centro Hospitalar de Trás-os-Montes e Alto Douro, Vila Real, Portugal

**Keywords:** conversion disorder, hysteria, stuttering, functional neurologic disorder

## Abstract

**Introduction:**

Conversion Disorder is a condition defined by the sudden appearance of neurologic symptoms without an identifiable organic cause, often thought to be associated with psychological triggers. This disorder can lead to severe distress and loss of functionality which, without appropriate treatment, can be made permanent.

**Objectives:**

To raise awareness for this unexplained and often misunderstood disorder using a clinical case as background.

**Methods:**

Clinical history, organic evaluation, psychological evaluation and literature review.

**Results:**

28-year-old female, single, with two children, working from home as a call-centre operator. Previously followed and medicated for depression. Presents to the ER due to sudden loss of consciousness while working, after which her speech became hindered by stuttering. Neurologic evaluation was unremarkable and she was referred for Psychiatric assessment, resulting in a diagnosis of Conversion Disorder. Speech was at first understandable but in the following weeks became progressively worse and eventually led to aphonia, while written communication remained normal and was the patient’s method of choice.

**Conclusions:**

Once a favourite of Psychiatrists, little is yet known about the underlying mechanisms behind this disorder. Experts disagree on whether to classify it as a dissociative disorder, a somatoform disorder, or its own category. Patients presenting with this condition are often mistaken for malingering and thus subject to unhelpful or outright discriminatory practices. Broadened awareness is required to ensure patients get early access to the best possible care and thus improve their quality of life.

